# Defluoridation of water through the transformation of octacalcium phosphate into fluorapatite

**DOI:** 10.1016/j.heliyon.2019.e02288

**Published:** 2019-08-14

**Authors:** Alfredo Idini, Elisabetta Dore, Dario Fancello, Franco Frau

**Affiliations:** Department of Chemical and Geological Sciences, University of Cagliari, 09042, Monserrato (CA), Italy

**Keywords:** Materials science, Materials synthesis, Environmental science, Environmental geochemistry, Environmental pollution, Water pollution, Water quality, Earth sciences, Water defluoridation method, OCP synthesis, OCP-FAP transformation, Dissolved fluoride removal

## Abstract

The consumption of water with fluoride concentration higher than 1.5 mg/L (WHO recommended limit) is recognized to cause serious diseases, and fluoride removal from natural contaminated waters is a health priority for more than 260 million people worldwide. The octacalcium phosphate (OCP), a mineralogical precursor of bio-apatite, is here tested as a fluoride remover. A new two-step method for the synthesis of OCP is proposed: 1) synthesis of brushite from calcium carbonate and phosphoric acid; 2) subsequent hydrolysis of brushite. Fluoride removal experiments are performed in batch-mode using different initial concentrations of fluoride (from 40 to 140 mg/L) and reaction times. Most of fluoride is removed within the first 2 h of all experiments, and the drinkable limit of 1.5 mg/L is reached within a minimum of 3 h for an initial fluoride concentration of 40 mg/L. The experimental fluoride removal capacity of OCP is 25.7 mg/g, and 4 g of OCP can effectively treat 1 L of water with fluoride concentration up to 50 times higher than the drinking limit of 1.5 mg/L. XRD and chemical characterization of the solid phases, before and after the removal experiments, indicate that OCP transforms into fluorapatite (FAP) uptaking fluoride from solution.

## 1 Introduction

**Fig. 1:**
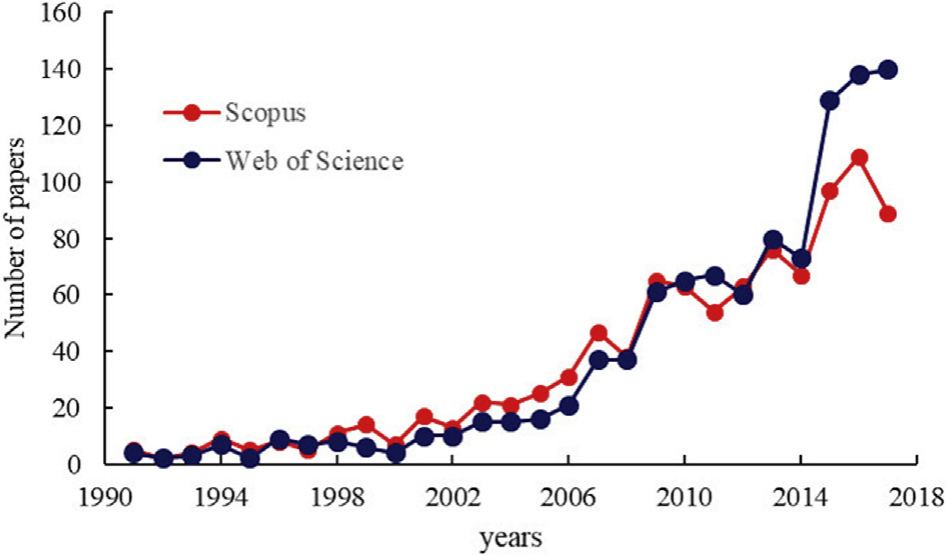
The diagram shows the trend of papers published in indexed journals from 1991 to 2108 in the topic of defluoridation of water. Sources: www.scopus.com (Scopus); www.webofknowledge.com (Web of Science).

Fluorine is an essential micronutrient for human health but the World Health Organization (WHO) recommends a maximum concentration of fluoride (F^−^) in drinking water equal to 1.5 mg/L (WHO, 2011) because higher F^−^ concentrations are known to cause many diseases, such as skeletal fluorosis and neurological issues (Ozsvath, 2009; Tewari and Dubey, 2009). Geogenic contamination of F^−^ in groundwater is one of the major problems of the water crisis of the 21^st^ century, affecting more than 260 million people over 24 countries worldwide. For instance, high concentrations of F^−^ in in sodium-bicarbonate groundwaters with average pH in the range of 8.0–8.5 have been reported in several rural areas along the East African Rift Valley (Ethiopia, Kenya, Tanzania) (Tekle-Haimanot et al., 2006; Ghiglieri et al., 2012; Olaka et al., 2016) and in different areas of Pakistan, India and China (Farooqi et al., 2007; Gao et al.*,* 2007; Singh et al., 2018). This is the reason why the online databases Scopus and Web of Science together report more than 1100 papers on the topic “water defluoridation”, of which 400 in the last 4 years (data updated to 02/11/2018) (Fig. 1). The most recent reviews (Ayoob and Gupta, 2006; Mohapatra et al., 2009; Bhatnagar et al., 2011; Renuka and Pushpanji, 2013; Vithanage and Bhattacharya, 2015; Singh et al., 2016; Yadav et al., 2018) indicate that several materials, both synthetic and natural, and technologies have been tested and proposed for the removal of F^−^ from water, and the mechanisms involved mainly are: ion exchange, precipitation, Donnan dialysis, electrodialysis, reverse osmosis, nanofiltration and adsorption. However, in agreement with Yadav et al. (2018), the removal of excess amounts of F^−^ from groundwater is still to be solved in rural areas of developing countries where people have insufficient access to drinking water and a defluoridation technique should be as much as possible: (i) simple; (ii) effective; (iii) based on cheap materials and devices; (iv) easy to use; (v) free of collateral effects on water quality.

This study, as a part of the *FLOWERED* project (a Horizon 2020 European funded project) aimed at developing a defluoridation method for drinking water suitable for rural areas of the East African Rift Valley (EARV) and with the above-mentioned features, presents a new method for water defluoridation through the use of octacalcium phosphate (OCP; Ca_8_(HPO_4_)_2_(PO_4_)_4_ ∙ 5H_2_O), based on the same mineralogical principle of accumulation of F^−^ into the hard tissues of human body. In fact, the inorganic part of animal and human bones and teeth consists of non-stoichiometric calcium-deficient carbonate hydroxyapatite, also called bio-apatite (Sakae et al., 2015), whose crystallization is subsequent to the formation of its natural precursor OCP (Suzuki, 2013; Lu et al., 2015). In case of long-term high F^−^ exposure, the F^−^ ion can be accumulated into the hard tissue by means of the formation of fluorapatite (FAP; Ca_5_(PO_4_)_3_F) instead of bio-apatite (Inoue et al., 2011).

The use of calcium phosphates, such as brushite (also known as dicalcium phosphate dihydrate – DCPD; CaHPO_4_ ∙ 2H_2_O) and hydroxyapatite (HAP; Ca_5_(PO_4_)_3_(OH)), for water defluoridation is widely studied, but information about the use of OCP is lacking. In fact, synthetic OCP is extensively used in biomedical applications for bone regeneration (Markovic and Chow, 2010; Suzuki, 2013) but at the best of our knowledge only Yang et al. (2012) investigated the F^−^ uptake capacity from water by OCP.

In this work the OCP effectiveness as F^−^ remover is tested through laboratory sorption experiments conducted at circum-neutral pH and room conditions, using synthetic aqueous solutions and varying some parameters such as the contact time and the initial F^−^ concentration, as well as using a natural aqueous matrix with addition of F^−^.

This work does not claim to be all-encompassing, and focuses exclusively on laboratory experiments aimed at understanding the F^−^ removal mechanism using OCP. Future studies will be more applicative and mainly devoted to: (i) test the removal capacity of OCP in presence of coexisting anions other than F^−^, also using different starting pH values; (ii) test OCP with natural fluoride-rich groundwaters from the EARV in order to obtain important information about the real application of OCP in water defluoridation.

## 2 Materials and methods

All the reagents used for the synthesis and the removal experiments were of analytical grade (Carlo Erba reagents ACS-for analysis) and without further purification; ultrapure water (Millipore, Milli-Q©, 18.2 MΩ cm) was used to dissolve the salts and to dilute the solutions.

### 2.1 Synthesis

The OCP was synthesized in two steps. Firstly, synthetic DCPD was synthesized at room temperature adding 0.366 mol of both H_3_PO_4_ and CaCO_3_ in 2 L of ultrapure water acidified at pH 1.5 with HCl. After precipitation, the DCPD was recovered through filtration, and dried at 40 °C. During the second step the OCP was obtained from the DCPD hydrolysis: 1.2 g of DCPD was added to 500 mL of ultrapure water with starting pH = 7.2; the solution was then heated in a stove at 60 °C for 65 h; the solid was recovered through filtration and dried at room temperature.

### 2.2 Fluoride removal experiments

Fluoride removal experiments were performed in batch-mode using 50 mL conical flasks agitated through a rotor system (40 rpm), at room temperature: 200 mg of OCP were added to 50 mL solutions (solid/solution ratio = 4 g/L) with different initial F^−^ concentrations (40, 60, 80, 120, 140 mg/L), for different times of reaction (from 0.5 to 21 h). Fluoride-bearing solutions were obtained dissolving NaF in ultrapure water stabilized at pH 8 using NaOH.

A test with a natural aqueous matrix was performed using 8 L of a low calcium, sodium-bicarbonate commercial bottled water, whose composition very well simulates fluoride-rich groundwaters from the EARV (Tekle-Haimanot et al., 2006). An adequate amount of NaF was added to the bottled water in order to have an initial F^−^ concentration of 30 mg/L. Before starting the experiment, 32 g of OCP were added to the solution reaching a solid/solution ratio of 4 g/L. The solution was kept under stirring at 500 rpm at room temperature (25 ± 3 °C) for 12 h inside a PE container with a capacity of 10 L.

All experiments were replicated twice or more. The solution pH of each experiment was measured before the addition of OCP and at the end of the reaction time. At the end of the experiments the solids and solutions were separated through filtration and recovered to carry out the mineralogical characterization and chemical analyses.

### 2.3 Chemical analyses and mineralogical characterization

The concentration of F^−^ in solution before and after the experiments was determined by a potentiometer (sensION TM + MM340, *HACH* LANGE) with Ion Selective Fluoride Electrode (ISE F^-^ 9655C, *HACH* LANGE) and adding the TISAB III (*HACH*) solution to avoid interference of metallic complexes during the analysis. A portion of OCP and the solid recovered after each experiment was dissolved in 5% v/v HNO_3_ and then diluted with ultrapure water for the chemical analyses. The concentrations of Ca and P in both solids and solutions were determined by inductively coupled plasma optical emission spectroscopy (ICP-OES, ARL Fisons 3520).

The cationic and anionic chemical composition of bottled water, before and after the F^−^ removal experiment, was analyzed by ion chromatography (IC); only P was measured by ICP-OES. The initial bicarbonate concentration was calculated by alkalinity determined with the Gran method; to obtain the final bicarbonate concentration the contribution of phosphate was subtracted from alkalinity.

The mineralogical characterization of DCPD, OCP and the solid recovered after each experiment was performed collecting XRD patterns in the 3.5–55 °2θ angular range on an automated PANalytical X'pert Pro diffractometer, with Ni-filtered Cu-K_α1_ radiation (λ = 1.54060 Å), operating at 40 kV and 40 mA, using the X'Celerator detector.

## 3 Results

### 3.1 Synthesis

**Fig. 2:**
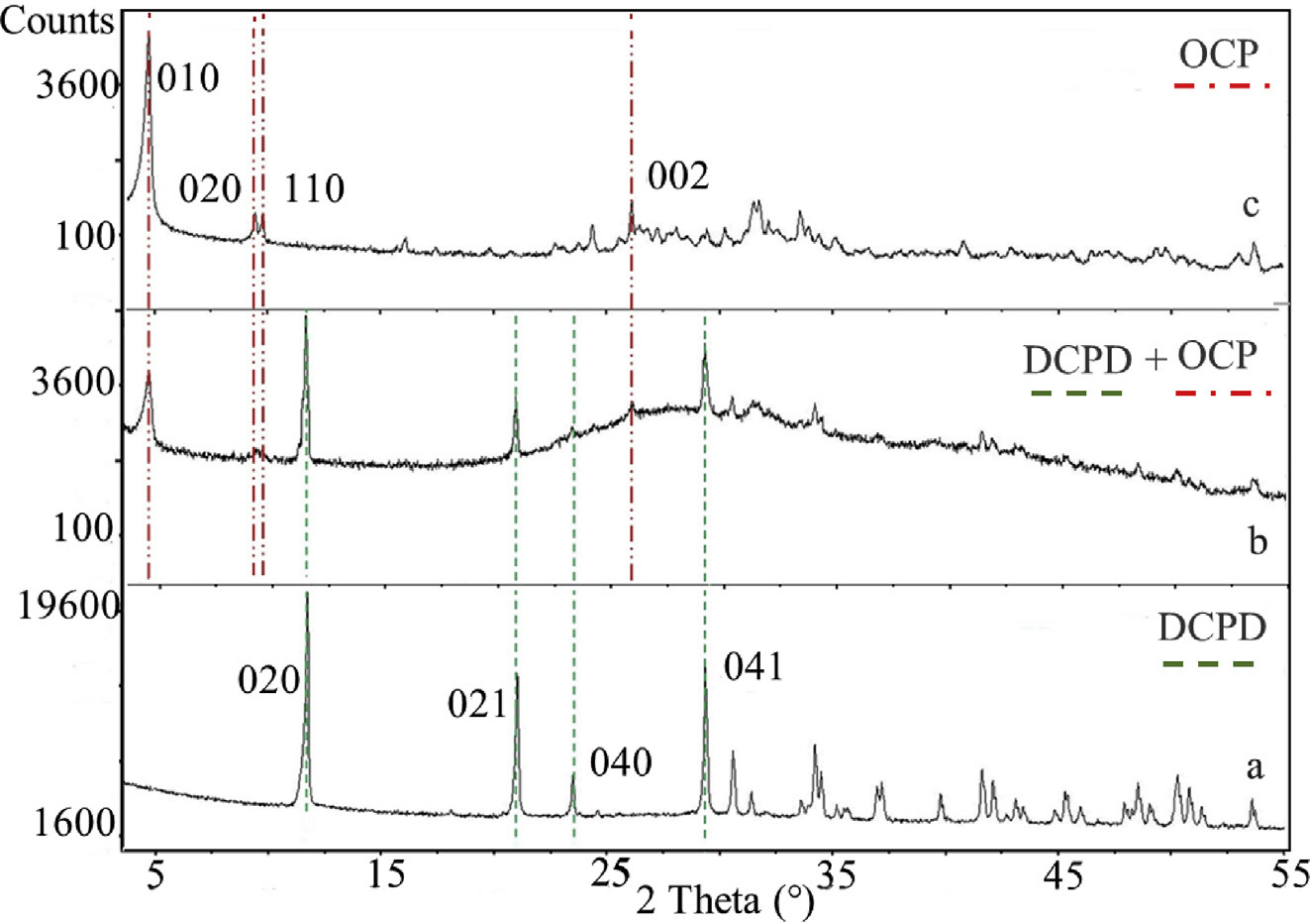
XRD patterns and labelled representative peaks of (a) DCPD, (b) its hydrolysis product after 40 h with initial appearance of OCP and (c) final transformation in OCP after 65 h at 60 °C. Reference ICSD patterns used for phase identification are n. 00-009-0077 for DCPD and n. 00-026-1056 for OCP.

The XRD pattern of the phase obtained from the first step of the synthesis reaction (Fig. 2a) shows that all the peaks are ascribable to DCPD (reference ICSD pattern n. 00-009-0077). The pattern of the product of DCPD hydrolysis after 40 h (Fig. 2b) still shows the presence of DCPD peaks and new peaks at 4.75, 9.41 and 9.73 °2θ corresponding to the (010), (110) and (020) characteristic reflections of OCP (reference ICSD pattern n. 00-026-1056), indicating that the hydrolysis reaction is in progress. The pattern of the solid recovered after 65 h (Fig. 2c) shows only the characteristic peaks of OCP, suggesting that the hydrolysis reaction has gone to completeness. Peaks of undesired phases were never detected.

The reaction of DCPD precipitation from aqueous solution and its hydrolysis into OCP can be summarized, respectively, by the reactions (1) and (2):

(1) CaCO_3_ + H_3_PO_4_ + 2 H_2_O → CaHPO_4_ ∙ 2H_2_O_(DCPD)_ + CO_2__(g)_ + H_2_O

(2) CaHPO_4_ ∙ 2H_2_O_(DCPD)_ ↔ 0.125 Ca_8_(HPO_4_)_2_(PO_4_)_4_ ∙ 5H_2_O_(OCP)_ + 0.25 HPO_4_^2-^ + 1.375 H_2_O + 0.5 H^+^

### 3.2 Sorption experiments

#### 3.2.1 Solutions

**Fig. 3:**
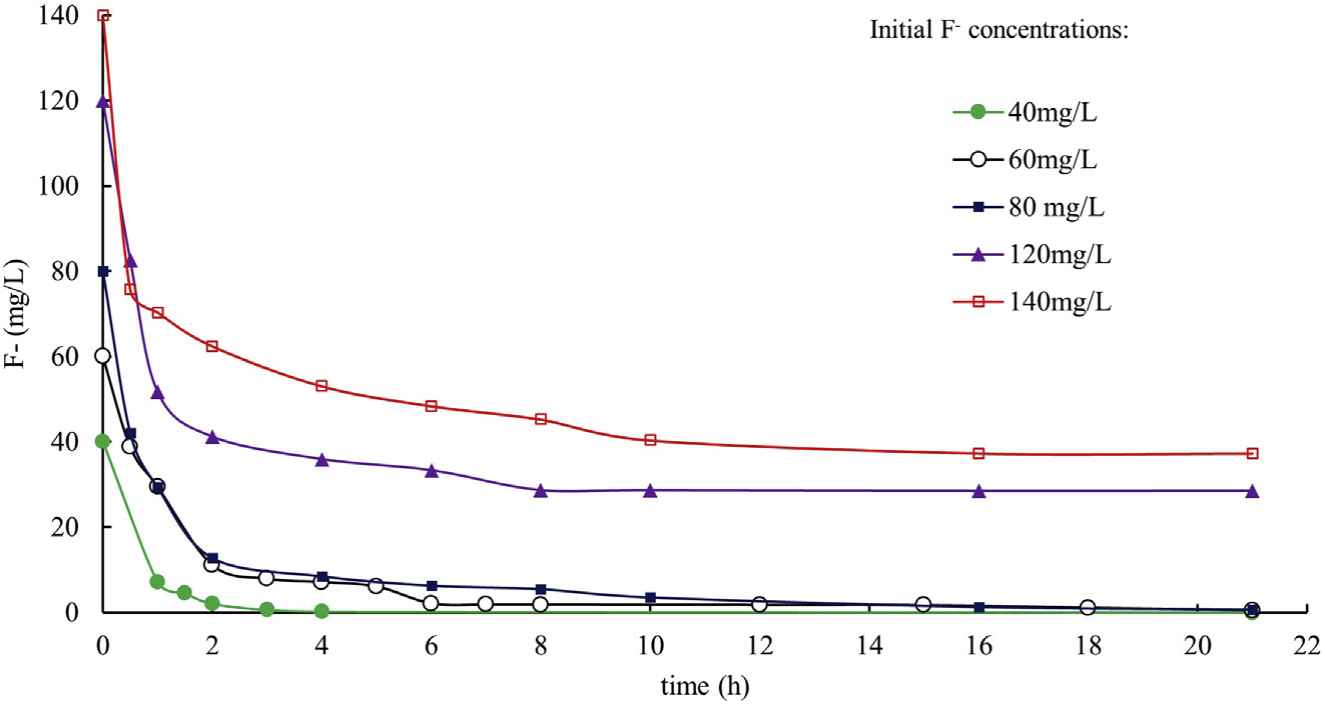
The diagram shows the removal trend of dissolved F^−^ by 200 mg of OCP in 50 mL of solution at different step times and different initial F^−^ concentrations.

The results of all sorption experiments show that most of F^−^ is removed from solution during the first hours after the addition of OCP, and the equilibrium is reached more o less quickly depending on the initial F^−^ concentration (F^−^ i.c.) (Fig. 3). The time required to reach the F^−^ equilibrium in solution varies from 3 h (F^−^ i.c. = 40 mg/L) to 10 h (F^−^ i.c. = 140 mg/L). More than 99% of F^−^ is removed in the experiments with F^−^ i.c. of 40, 60 and 80 mg/L, whereas the removal reaches 76% and 73% for F^−^ i.c. of 120 and 140 mg/L, respectively (Table 1). At the end of all experiments, an increase of dissolved P^5+^ (up to 72.4 mg/L) and Ca^2+^ (up to 0.26 mg/L) was observed, as well as a pH decrease from 8.0 to 6.5–7.0 (Table 1).

**Table 1:** Chemical parameters measured before (0 h) and at the end (21 h) of F^−^ removal batch experiments performed with 200 mg of OCP in 50 mL of solution. F^−^ i.c. = initial fluoride concentration in solution.

Experiment	F^−^ i.c. (0 h)	F^−^ (21 h)	F^−^ removed	Ca^2+^ (21 h)	total P^5+^ (21 h)	pH	
	mg/L	mg/L	%	mg/L	mg/L	0 h	21 h
Exp1	40	0.03	99.9	0.24	42.5	8	6.52
Exp2	60	0.53	99.1	0.26	44.6	8	6.62
Exp3	80	0.71	99.1	0.19	69.0	8	6.72
Exp4	120	28.5	76.2	0.16	72.4	8	6.92
Exp5	140	37.3	73.4	0.18	70.8	8	6.96

The results of the experiment with the bottled water (Table 2) show that dissolved F^−^ decreases from 30.4 to 0.18 mg/L, well below the drinking limit of 1.5 mg/L; other compositional differences before and after the treatment with OCP regard the decrease of dissolved Ca^2+^, Mg^2+^ and HCO_3_^-^, and the increase of P^5+^.

**Table 2:** Compositional differences of the bottled water before and after the F^−^ removal experiment.

	pH	Conductivity	T	Na^+^	K^+^	Mg^2+^	Ca^2+^	P^5+^_tot_	F^-^	Cl^-^	NO_3_^-^	SO_4_^2-^	HCO_3_^-^
		μS/cm	°C	mg/L	mg/L	mg/L	mg/L	mg/L	mg/L	mg/L	mg/L	mg/L	mg/L
Initial composition	8.3	596	25.2	123	1.6	3.9	12.2	0	30.4	79	5.0	16.1	126
Final composition	7.4	506	24.6	122	1.7	0.1	0.20	46.2	0.18	79	2.4	15.8	28.2

#### 3.2.2 Solids

**Fig. 4:**
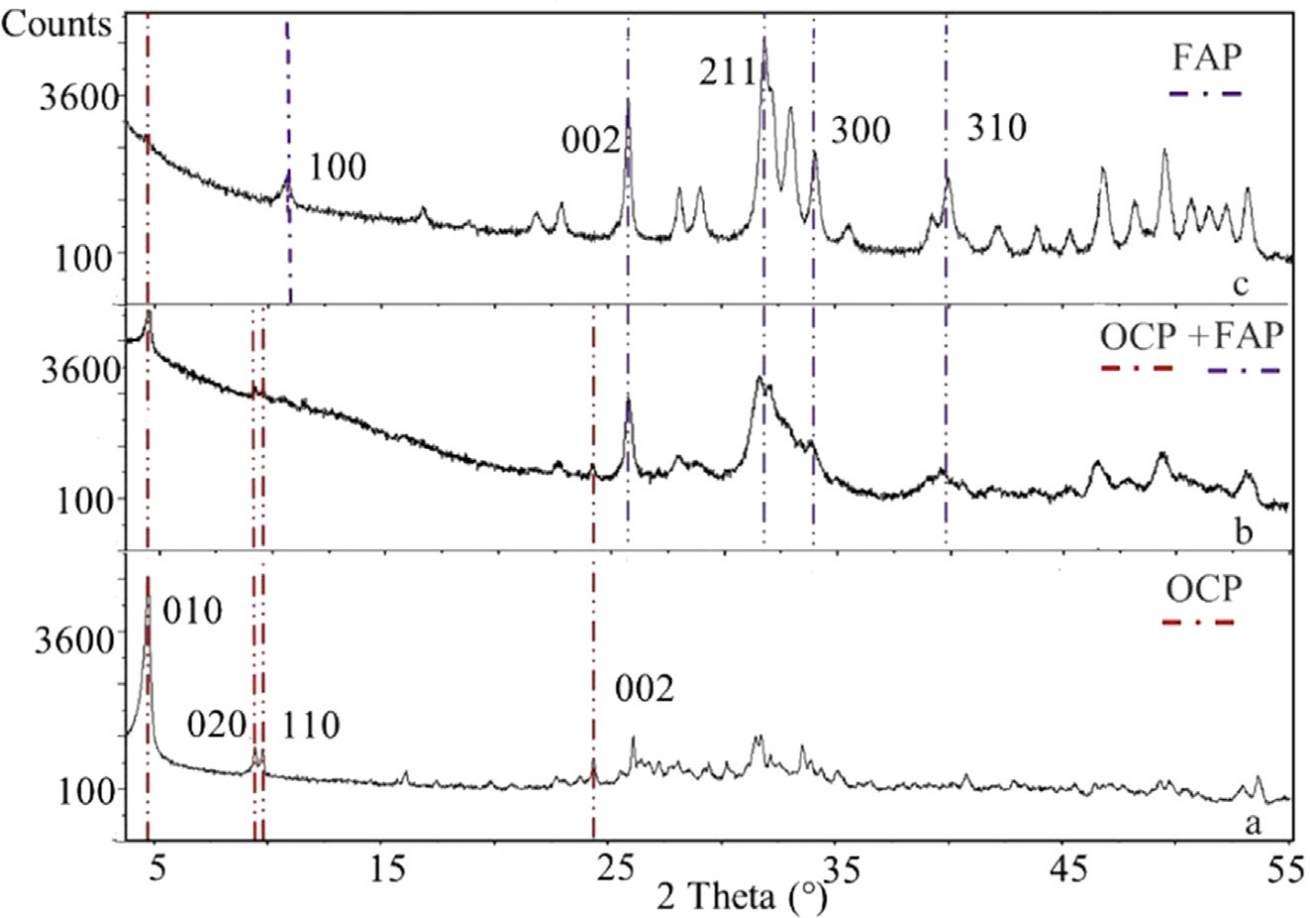
XRD patterns of a) OCP that is the initial phase before F^−^ removal experiments; b) solid phase recovered after the experiment with F^−^ i.c. (initial fluoride concentration in solution) = 40 mg/L; c) solid phase recovered after the experiment with F^−^ i.c. = 140 mg/L. Phases shown in XRD patterns b) and c) were collected after 21 h of contact time with F^−^ solutions. Reference ICSD pattern used for identification of FAP is n. 00-015-0876.

The XRD patterns of synthetic OCP and solids recovered at the end of the experiments with F^−^ i.c. of 40 and 140 mg/L are shown in Fig. 4. The solid phases after F^−^ uptake point out the transformation of OCP into FAP. The transformation is partial for the experiment with the lowest F^−^ i.c. (40 mg/L) whose XRD pattern shows the peaks of both phases, whereas the transformation appears to be complete for the experiment with the highest F^−^ i.c. (140 mg/L) when only the characteristic peaks of FAP are visible in the corresponding XRD pattern (Fig. 4). The XRD patterns of the experiments with intermediate F^−^ i.c. (not reported in Fig. 4) mainly show the presence of FAP, with OCP barely detectable.

The certain presence of FAP, that cannot be only assessed on the basis of XRD analysis due to the very similar XRD patterns of all end-members of the apatite series, is confirmed by the results of chemical analyses performed on the solid phases recovered after the sorption experiments. In fact, the amount of F^−^ in the solids increases as F^−^ i.c. increases (Table 3). Moreover, the molar Ca/P ratio of the phases recovered after the experiments with F^−^ i.c. of 80, 120 and 140 mg/L (Ca/P = 1.62, 1.62 and 1.64, respectively) is very close to the theoretical one for FAP (Ca/P = 1.67), whereas it is significantly lower when OCP is still detectable (Fig. 4b) at the end of the experiments with F^−^ i.c. of 40 and 60 mg/L (Ca/P = 0.93 and 1.14, respectively) (Table 3).

**Table 3:** Chemical composition of solid phases recovered after F^−^ removal experiments.

Experiment	F^−^ i.c. (0 h)	Detected phases	Ca^2+^	total P^5+^	F^-^	Ca^2+^/P^5+^	
	mg/L		wt.%	wt.%	wt.%	mass ratio	molar ratio
Exp1	40	OCP + FAP	29.6	24.6	0.8	1.20	0.93
Exp2	60	OCP + FAP	31.5	21.3	1.1	1.48	1.14
Exp3	80	FAP + OCP	37.6	17.9	1.6	2.10	1.62
Exp4	120	FAP	37.2	17.8	3.1	2.09	1.62
Exp5	140	FAP	38.3	18.1	3.4	2.12	1.64
OCP theoretical			35.9	20.8	0	1.73	1.33
FAP theoretical			39.7	18.4	3.77	2.16	1.67

## 4 Discussion

### 4.1 Water defluoridation

The experimental data show that 200 mg of OCP in 50 mL of deionized water can reduce the F^−^ content from initial concentrations of 40, 60 and 80 mg/L to concentrations lower than the WHO limit for drinking water of 1.5 mg/L (Table 1). This result means that 4 g of OCP can effectively treat 1 L of water with F^−^ concentration up to 50 times higher than the drinking limit of 1.5 mg/L. Importantly, no deleterious collateral effects on the quality of the treated water are produced during the defluoridation process: the solution pH slightly decreases, however remaining in the range suggested by WHO for drinking water (pH = 6.5–9.5), and the increase of dissolved phosphorus (Table 1) can be considered a positive effect as phosphorus is an essential element for life (U.S National Academy of Science, 1997) not subject to limitations for drinking water (WHO, 2011).

The effectiveness of the method in removing F^−^ from water is confirmed also using a natural aqueous matrix (Table 2), where the observed compositional variations are related to the F^−^ removal mechanism.

Furthermore, both physical (pH, conductivity, etc.) and organoleptic (taste, turbidity, etc.) features of the water defluoridated with the proposed method meet the WHO requirements.

### 4.2 Removal mechanism

The chemical and mineralogical results indicate that the F^−^ removal from aqueous solution takes place by means of the transformation of OCP into FAP. This reaction can be schematized as follows:

(3) Ca_8_(HPO_4_)_2_(PO_4_)_4_ ∙ 5H_2_O_(OCP)_ + 1.6 F^−^ → 1.6 Ca_5_(PO_4_)_3_F_(FAP)_ + 1.2 HPO_4_^2-^ + 5 H_2_O + 0.8 H^+^

It is known that the transformation of OCP into HAP is controlled by hydrolysis and subsequent epitaxial growth of HAP on OCP (Zhan et al., 2005; Arellano-Jiménez et al.*,* 2009; Carino et al., 2018), with a minor contribution from OCP dissolution and HAP precipitation that must be taken into account. Considering that FAP and HAP have a complete anion solid solution (i.e. F^−^ ↔ OH^−^) (Hughes and Rakovan, 2002), we assume the same mechanism of transformation from OCP into FAP.

**Fig. 5:**
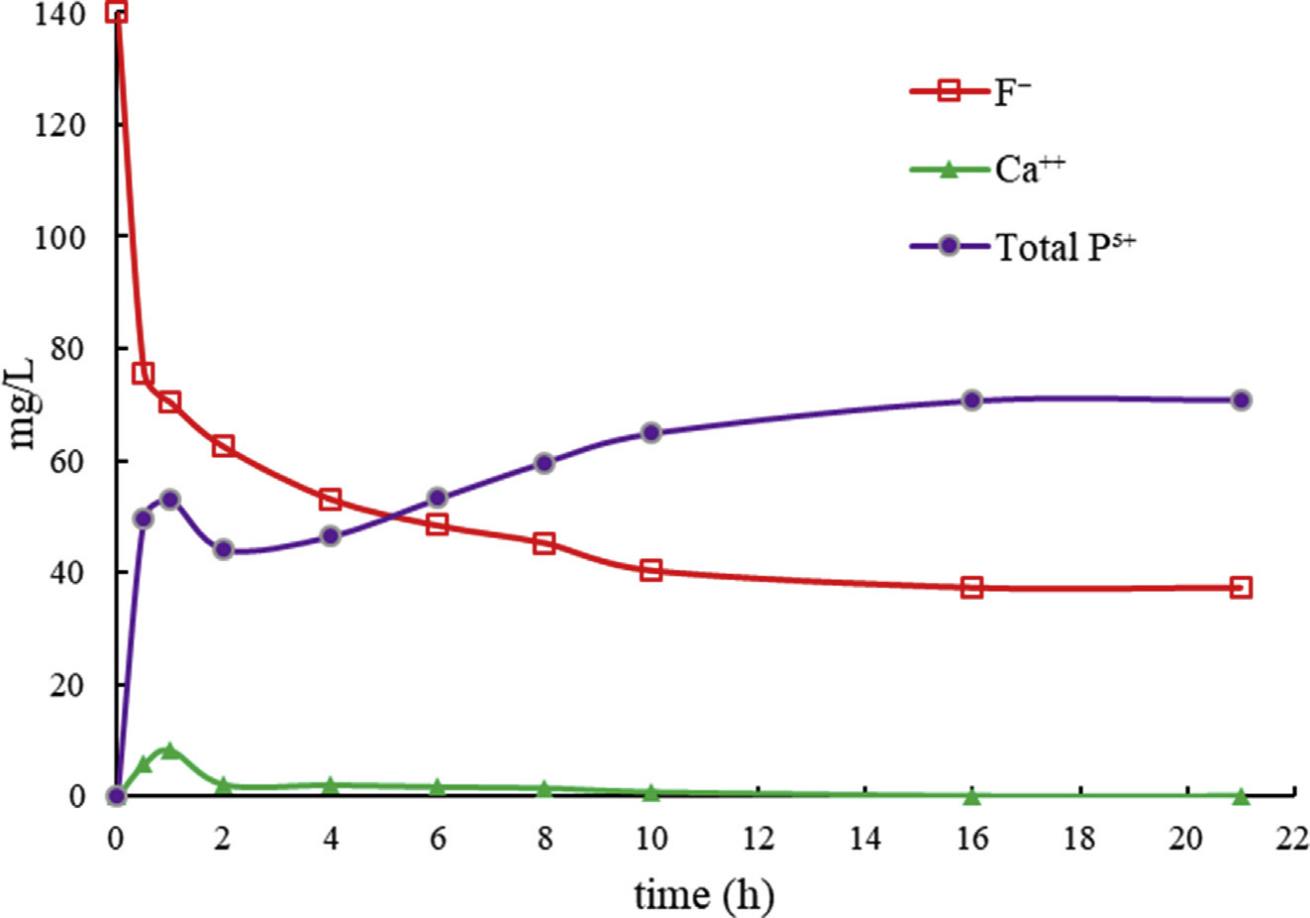
The diagram shows the concentrations of calcium, total phosphorus and fluoride as a function of time, during the F^−^ removal experiment with OCP and F^−^ i.c. = 140 mg/L.

The contribution of both reactions (i.e. OCP hydrolysis and FAP growth) can be evaluated monitoring the concentrations of Ca^2+^, P^5+^ and F^−^ in solution during the experiments. The concentrations in solution of these elements as a function of time, during the representative experiment with F^−^ i.c. = 140 mg/L, are shown in Fig. 5. The sharp increase of P^5+^ and partly of Ca^2+^ in solution, occurring within the first hour simultaneously with the sharp decrease of F^−^, is related to the solubility of OCP, that is about 8 mg/L (Dorozhkin, 2012), and the consequent oversaturation and precipitation of FAP which has a solubility of about 0.2 mg/L (Dorozhkin, 2012). After about 1.5 h, the concentration of Ca^2+^ in solution decreases below 0.2 mg/L, while P^5+^ continues to increase; this can be explained with transformation of OCP into FAP that has a molar Ca/P ratio (1.67) higher than that of OCP (1.33), with consequent release of phosphate to solution.

In the solid phases recovered after the experiments with F^−^ i.c. of 120 and 140 mg/L, where the transformation OCP → FAP appears to be complete, the molar Ca/P ratio is lower than the stoichiometric value of theoretical FAP (Table 3). The deficiency of Ca in the minerals of the apatite series often occurs in natural apatites and can be explained with the substitution of PO_4_^3-^ by HPO_4_^2-^ (Hutchens et al., 2006) and/or CO_3_^2-^ (Vallet-Regí and González-Calbet, 2004). These substitutions cause the decrease of the negative charge, and therefore the amount of Ca necessary to counterbalance can decrease too. The chemical analyses confirm that in every experiment the formed apatite is mainly FAP and that its formation depends on the initial F^−^ concentration available in solution. In fact, the apatite obtained at the end of the experiments could be defined as a calcium-deficient fluoro-hydroxyl-apatite.

The F^−^ removal mechanism is confirmed also in the experiment with the bottled water (Table 2). In fact, the increase of P^5+^ and the decrease of Ca^2+^ in solution are explainable by the higher molar Ca/P ratio in FAP (1.67) with respect to the starting molar Ca/P ratio in OCP (1.33), whereas the decrease of HCO_3_^-^ can be explained by (i) lowering of pH from 8.3 to 7.4, and (ii) replacement of PO_4_^3-^ with carbonate species into the FAP structure.

### 4.3 Modeling

The empirical F^−^ removal capacity has been calculated with the formula:

(4) *Q*_*t*_*= (C*_*i*_*– C*_*t*_*) V / W*

where the removal capacity *Q*_*t*_ is the F^−^ removed per unit of sorbent (mg/g) at the *t* time (h), *C*_i_ and *C*_*t*_ are, respectively, the initial F^−^ concentration and the F^−^ concentration at *t* time in solution (mg/L), *V* is the volume of solution (L) and *W* is the weight of sorbent (g).

The experimental removal capacity of OCP calculated at the equilibrium (*Q*_*exp*_) (Table 4) increases as the initial F^−^ concentration increases, and reaches 25.7 mg/g in the experiment with F^−^ i.c. = 140 mg/L. This value is lower than the stoichiometric theoretical F^−^ uptake capacity of reaction (3) (34.0 mg/g) calculated considering that 1 mol of OCP (molar mass 892.468 g/mol) reacts with 1.6 mol of F^−^ (molar mass 18.998 g/mol) to form FAP. The lower value of measured F^−^ uptake can be explained considering: (i) the partial ingress of OH^−^ instead of F^−^ in the FAP lattice; (ii) the Ca-deficiency of FAP (Table 3); the slight solubility of FAP (Dorozhkin, 2012).

**Table 4:** Fluoride sorption capacity of OCP determined at equilibrium from the experimental data (*Q*_*exp*_) and calculated from the pseudo-second order kinetic model (*Q*_*e*_), and the sorption Langmuir isotherm parameters calculated using experimental data at equilibrium (21 h).

Experiment	F^−^ i.c. (0 h)	F^−^ (21 h)	Experimental	Pseudo-second order kinetics			Langmuir isotherm		
			*Q*_*exp*_	*Q*_*e*_	*k*	R^2^	*Q*_*max*_	*K*_*L*_	R^2^
	mg/L	mg/L	mg/g	mg/g	g/mg h		mg/g	L/mg	
Exp1	40	0.03	9.99	10.1	0.0138	0.9999			
Exp2	60	0.53	14.9	15.2	0.0577	0.9989			
Exp3	80	0.71	19.8	19.6	0.0241	0.9998			
Exp4	120	28.5	22.9	23.5	0.0192	0.9995			
Exp5	140	37.3	25.7	26.6	0.0260	0.9987			
							26.8	0.7645	0.9953

Although the removal mechanism of F^−^ from solution by using OCP has been demonstrated not to be adsorption but precipitation and epitaxial growth of FAP at the expense of dissolving OCP, the application of the Langmuir isotherm to our results can be useful as a simple interpolation model to compare stoichiometric and empirical removal capacity values with calculated one (i.e., *Q*_*max*_*)*. The theoretical maximum sorption capacity (*Q*_*max*_) has been calculated through the linear form of the Langmuir isotherm equation expressed as follows:

(5) *C*_*e*_*/Q*_*exp*_*= (1/K*_*L*_*Q*_*max*_*) + (1/Q*_*max*_*) C*_*e*_

where *C*_*e*_ is the F^−^ concentration in solution at equilibrium (mg/L), *Q*_*max*_ is the maximum theoretical sorption capacity (mg/g), and *K*_*L*_ is the Langmuir constant (L/mg).

**Fig. 6:**
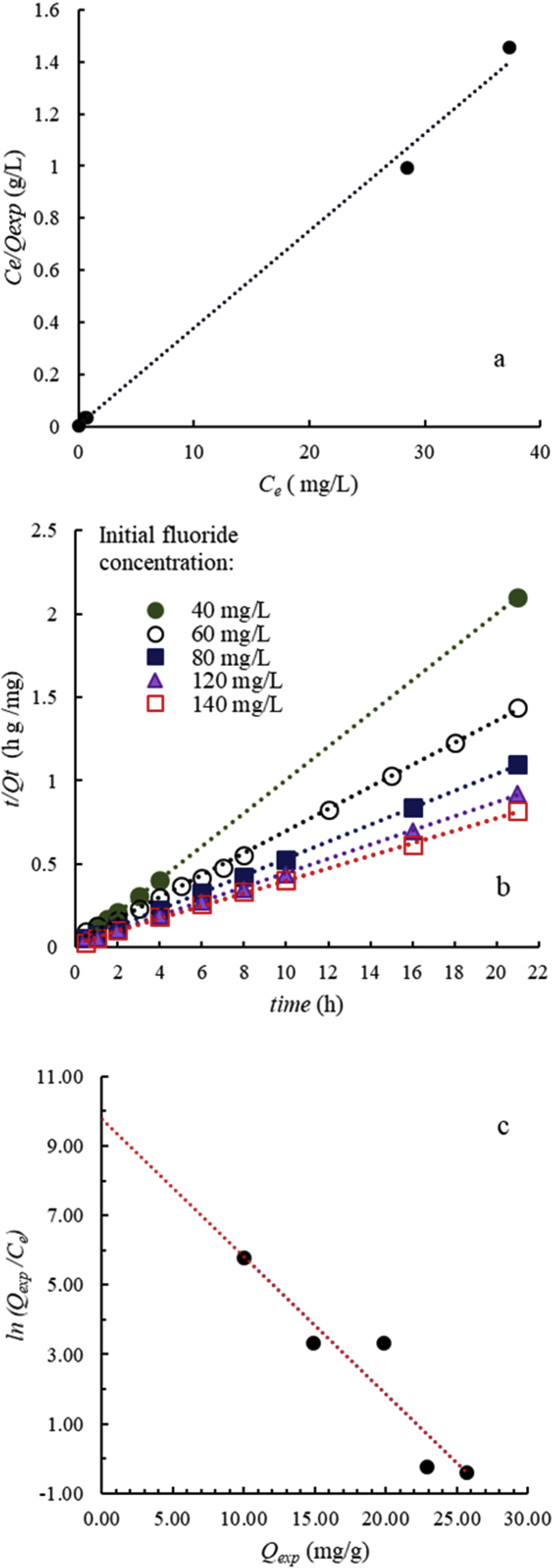
Diagrams of a) plot of the linearized Langmuir isotherm applied to F^−^ removal from solution by OCP, calculated using experimental data at equilibrium (21 h); b) plot of the linearized pseudo-second order kinetic model for experiments performed with different initial F^−^ concentrations (60, 80, 120, 140 mg/L); c) plot of *ln(Q*_*exp*_*/C*_*e*_*)* vs *Q*_*exp*_ for F^−^ removed from solution by the hydrolysis reaction OCP → FAP.

The plot of *C*_*e*_*/Q*_*exp*_ vs *C*_*e*_ shows a good linearity (Fig. 6a), therefore the maximum sorption capacity (*Q*_*max*_) and the Langmuir constant *K*_*L*_ can be calculated from the slope (*1/Q*_*max*_) and from the intercept (*1/K*_*L*_ *Q*_*max*_) of the straight line in the plot of Fig. 6a. The *Q*_*max*_ value is 26.8 mg/g, that is rather close to the *Q*_*exp*_ (25.7 mg/g) obtained with F^−^ i.c. of 140 mg/L (Table 4).

The effect of contact time has been studied through the pseudo-second order kinetic equation (Ho and McKay, 1999) expressed as:

(6) *dQ*_*t*_*/dt = k(Q*_*e*_*- Q*_*t*_*)*^*2*^

where *k* is the rate constant of pseudo-second order sorption (g/mg h) and *Q*_*e*_ is the calculated F^−^ removal capacity at equilibrium (*Q*_*e*_*= Q*_*exp*_). The integration of Eq. (6) written in linear form is:

(7) *t/Q*_*t*_*= (1/kQ*_*e*_^*2*^*) + (1/Q*_*e*_*)*_*t*_

The applicability of the pseudo-second order kinetic model on the sorption system is indicated by the linear relationship of the plot of *t/Q*_*t*_ vs *t* (Fig. 6b), that can be used to calculate the F^−^ removal capacity at equilibrium (*Q*_*e*_*)* and *k* from the straight line slope (1/*Q*_*e*_) and the intercept (1/*kQ*_*e*_^2^), respectively (Table 4). The *Q*_*e*_ values are very close to the corresponding *Q*_*exp*_, and in particular the *Q*_*e*_ value obtained with F^−^ i.c. of 140 mg/L (26.6 mg/g) is almost identical to *Q*_*max*_ (Table 4).

The Standard Gibbs free energy of formation (*ΔG°*) of reaction OCP → FAP can be calculated from the isotherm and is used to determine whether the reaction is spontaneous or not. The *ΔG°* is calculated from the following equation:

(8) *ΔG° = -RTlnK*_*o*_

where *R* is the universal gas constant (8.134 J/mol K), *T* is the temperature in Kelvin (293.15 K = 20 °C) and *K*_*o*_ is the dimensionless equilibrium constant of the reaction defined as:

(9) *K*_*o*_*= a*_*s*_*/a*_*e*_*= (v*_*s*_*Q*_*exp*_*)/ (v*_*e*_*C*_*e*_*)*

where *a*_*s*_ is the activity of F^−^ in the solid, *a*_*e*_ is the activity of F^−^ in the aqueous solution at equilibrium, and *v*_*s*_ and *v*_*e*_ are their relative activity coefficients. If the F^−^ concentration in solution decreases and approaches to zero, the activity coefficient *v* approaches to the unit and Eq. (9) can be written as follows:

(10) *lim* = *a*_*s*_*/a*_*e*_*= Q*_*exp*_*/C*_*e*_*= K*_*o*_

Q_exp_ → *0*

According to the method used by various authors (Khan and Singh, 1987; Gunay et al., 2007), *K*_*o*_ can be determined also by plotting *ln(Q*_exp_*/C*_*e*_*)* vs. *Q*_*e*xp_ (Fig. 6c). The value of Y-axis of *C*_*e*_ interpolated at zero corresponds at *lnK*_*o*_ = 9.76, and therefore the value of *ΔG°* at 293 K is -23.3 kJ/mol. The calculated value of *ΔG°* is in agreement with that reported for the formation of apatite (Drouet, 2015), and indicates that the reaction of transformation of OCP into FAP is spontaneous at room conditions.

### 4.4 Comparison with other methods based on calcium phosphates

One of the most studied and used defluoridation methods is based on the OH^−^ ↔ F^−^ ion exchange through HAP that, in presence of F^−^ in solution, forms FAP (Waghmare and Arfin, 2015). The use of HAP has some advantages, given that the method does not produce negative effects on treated water and its application is cheap and low-tech compared with other defluoridation methods. HAP can be obtained via different processes: (i) synthesis, but this method increases the cost of production; (ii) extraction from calcium phosphate-bearing rocks, but these source-rocks commonly host FAP and undesired elements for human health, such as uranium and other heavy metals (Abouzeid, 2008); (iii) calcination of animal bones (bone char), but this method requires a large amount of bones and a correct calcination temperature. Furthermore, the latter method, which is by far the most widely used, requires: (i) a control on the size of bone char particles and relative filter pores to avoid the release of very fine bone char particles into the treated water; (ii) a sanification process to avoid the bad taste and smell of water after the treatment, as well as the proliferation of bacteria inside the filter (Smittakorn et al., 2010).

In relatively recent years, other synthetic calcium phosphate compounds have been tested for defluoridation of water. The comparison of the different materials is a hard task to deal with because they were tested in different experimental conditions (e.g., initial F^−^ concentration, pH, solid/liquid ratio, etc.). For this reason, the different materials and their F^−^ removal capacity reported in Table 5 were selected according to the following criteria: (i) no pH buffering during the F^−^ removal experiment, and initial and final pH values in the range of drinking water (pH = 6.5–9.5); (ii) the lowest initial F^−^ concentration higher than 5 mg/L; (iii) absence or minimization of negative effects on the overall quality of treated water; (iv) experimental temperature close to 20–30 °C. The aim of these criteria is not only to compare the F^−^ removal capacity of different calcium phosphate materials, but also to compare their possible applicability.

**Table 5:** Comparison of the F^−^ removal capacities of various calcium phosphate materials.

Materials	Abbreviation in the cited paper	*Q*_*exp*_	*Q*_*max*_	Reference
		mg/g	mg/g	
Hydroxyapatite	HAP	13.2	16.4	(Nie et al., 2012)
Hydroxyapatite	HAP	19.0		(Badillo-Almaraz et al., 2007)
Hydroxyapatite-nanowire	HAP	6.28	25.5	(He et al., 2016)
Hydroxyapatite	PHA	11.5		(Wagutu et al., 2018)
Brushite	DCPD	18.1		(Yang et al., 2012)
Brushite	DCPD	2.85	6.59	(Mourabet et al., 2011)
Brushite	CHA	18.2		(Wagutu et al., 2018)
Apatitic tricalcium phosphate	TCP	7.8	13.9	(Mourabet et al., 2012)
Octacalcium phosphate	OCP	22.7		(Yang et al., 2012)
Bone char	BC 400°	3	3.51	(Mbabaye et al., 2017)
Bone char	--	4.1	5.44	(Medellin-Castillo et al., 2007)
Monetite	DCP	26	66.7	(Yang et al., 2018)
Monetite	MONs@CS	2.87	50.0	(Shen et al., 2016)
Monetite	--	56.6	120	(Shen et al., 2018)
Octacalcium phosphate	OCP	25.7	26.8	Present study

As far as we know, the only paper that explores the uptake of F^−^ from water by OCP is the work of Yang et al. (2012) that compares the different kinetics of F^−^ uptake (and not the removal capacity at equilibrium) by different calcium phosphate materials (HAP, DCPD and OCP), both in absence and in presence of polymeric additives. In that work the reaction kinetics of F^−^ removal is in the following order: DCPD > OCP > HAP. The experiments were performed using only one initial F^−^ concentration of 50 mg/L in 20 mL of water and only one dosage of sorbent, that is different for each material (26.4 mg of HAP, 35.2 mg of OCP and 45.3 mg of DCPD). The value of removal capacity (mg/g), neither empirical nor calculated, is not discussed but we extrapolated it from the plots included in the paper (22.7 mg/g in Table 5).

## 5 Conclusions

The use of octacalcium phosphate (OCP) has been tested for the removal of fluoride (F^−^) from water in order to subsequently develop a simple, effective and low-cost defluoridation method suitable for rural communities affected by fluorosis in the East African Rift Valley (EARV) and in other parts of the world.

The synthesis method of OCP has been successfully realized using calcium carbonate and phosphoric acid, in order to lower the production costs using non-expensive reagents and avoid any possible negative secondary effect in terms of water drinkability. The results of experiments indicate that OCP can act as an effective remover of F^−^ from water via transformation into fluorapatite (FAP), obtaining experimental and calculated removal capacity of *Q*_*exp*_ = 25.7 mg/g and *Q*_*max*_ = 26.8 mg/g, respectively. Moreover, the release of some phosphorus to water during the transformation of OCP into FAP may result in an improvement of the quality of water after the treatment, because the minimum intake of phosphorus is 100 mg/die for infants, 700 mg/die for pregnant women, and 1250 mg/die for children and adults (U.S. National Academy of Science, 1997). Thus, drinking 2 L/day of water defluoridated with OCP can provide a significant percentage of phosphorus RDA (Recommended Dietary Allowance) to people living in rural areas in developing countries.

Further studies will be devoted to test the defluoridation performance of the OCP transformation into FAP in presence of different concentrations of other anions, such as Cl^−^ and HCO_3_^-^, and with different starting pH values. Testing OCP with natural fluoride-rich waters from the EARV will provide important information about the real application of OCP in water defluoridation in terms of appropriate dose of OCP and defluoridation time required. Finally, a defluoridator device based on OCP will be designed with low-cost and low-tech features in order to be easily applicable and usable in rural areas of the EARV.

## Declarations

### Author contribution statement

Alfredo Idini: Conceived and designed the experiments; Performed the experiments; Analyzed and interpreted the data; Contributed reagents, materials, analysis tools or data; Wrote the paper.

Elisabetta Dore: Performed the experiments; Analyzed and interpreted the data; Contributed reagents, materials, analysis tools or data.

Dario Fancello: Performed the experiments.

Franco Frau: Conceived and designed the experiments; Analyzed and interpreted the data; Wrote the paper.

### Funding statement

This work is a part of the Ph.D. project of Idini Alfredo Idini, supported by MIUR (Italian Ministry of Education, University and Research) in the frame of the Italian National Program PON R&I 2014–2020 “Innovative doctorates with industrial characterization”. This work is also supported by the FLOWERED project (Coordinator G. Ghiglieri), a Horizon 2020 European funded project (Grant Agreement - N. 690378) (www.floweredproject.org).

### Competing interest statement

The authors declare no conflict of interest.

### Additional information

No additional information is available for this paper.
